# How sociodemographic factors relate to trust in artificial intelligence among students in Poland and the United Kingdom

**DOI:** 10.1038/s41598-024-80305-5

**Published:** 2024-11-20

**Authors:** Jarosław Kozak, Stanisław Fel

**Affiliations:** Institute of Sociological Sciences, Faculty of Social Sciences, The John Paul II Catholic University of Lublin, al. Raclawickie 14, 20-950 Lublin, Poland

**Keywords:** Trust in artificial intelligence, Sociodemographic factors, UTAUT, Cultural differences, Students’ perspectives on AI, Information technology, Human behaviour

## Abstract

The article aims to determine the sociodemographic factors associated with the level of trust in artificial intelligence (AI) based on cross-sectional research conducted in late 2023 and early 2024 on a sample of 2098 students in Poland (1088) and the United Kingdom (1010). In the times of AI progressively penetrating people’s everyday life, it is important to identify the sociodemographic predictors of trust in this increasingly dynamically developing technology. The theoretical framework for the article is the extended Unified Theory of Acceptance and Use of Technology (UTAUT), which highlights the significance of sociodemographic variables as predictors of trust in AI. We performed a multivariate ANOVA and regression analysis, comparing trust in AI between students from Poland and the UK to identify the significant predictors of trust in this technology. The significant predictors of trust were nationality, gender, length of study, place of study, religious practices, and religious development. There is a need for research into the sociodemographic factors of trust in AI and for expanding the UTAUT to include new variables.

## Introduction

Artificial intelligence (AI) is becoming an increasingly omnipresent element of modern life, capable of transforming the way people live and work. The increasingly widespread use of AI is a process full of challenges, including those connected with individuals’ trust in this new technology^1^. Trust is a condition for AI to enjoy social acceptance and the basis enabling people to take full advantage of its capabilities^2^. With the rapid development of modern AI-based technologies, various sectors – from medicine, through finance and law, to industry—are intensively striving to win individuals’ trust as a key factor in the implementation of such advanced tools^3^.

There are many ways of defining AI, depending on the area of application and research perspective. In broad terms, AI can be defined as “the tangible real-world capability of non-human machines or artificial entities to perform, task solve, communicate, interact, and act logically as it occurs with biological humans”^4^. The basic tasks of such systems include imitating human intelligence by collecting and interpreting data, drawing conclusions, and processing information to optimize decision-making processes^5^. AI is an umbrella term for a variety of systems based on diverse and complex algorithms^6^. The mechanisms governing the operation of these systems often remain unclear to individuals, and the AI “decision-making” procedures are not always fully comprehensible^7^. This makes trust in AI a complex and at the same time intriguing research topic.

Today, trust is a complex and multidimensional category, linked with culture, collective mentality, morality, and the nuances of interpersonal relations^8^. Although the concept of trust is understood and defined in many different ways, it can be described as a kind of hope or faith that the trusting person has, his or her belief and sometimes even certainty regarding the future behaviours or states of the object of trust^9^. Trust in a technology manifests itself in people’s willingness to accept the impact of that technology, which results from its usefulness, the possibility of predicting the effects of its operation, and a belief in the reliability of the providers of modern solutions^10^. Therefore, trust in a technology can be defined as the belief that the technology will function predictably and reliably, leading to what the individual subjectively perceives as positive outcomes ^6,11^. In this context, trust in AI emerges as a category extremely important and fascinating from the cognitive perspective.

The literature on the subject distinguishes six groups within the category of trust in modern technologies: transparency and explainability, accuracy and reliability, automation, anthropomorphism, mass data extraction, and experience and familiarity with the system. Taken together, these make up a complex picture of trust in the context of interaction with modern technologies^12^. What is equally important is personal predispositions to use such technologies, associated with individuals’ social and demographic characteristics. The factors of significance for individuals’ trust in AI also include issues such as prior experience, openness to novelties, constant pursuit of new knowledge, and striving to develop one’s skills^13^.

In modern societies, an important social category is students. They will be the social elite in the future, performing functions in various spheres of socioeconomic life and state administration. They are also a social group open to technological innovations, have relatively extensive knowledge about them, and gladly use them^14^. The growing interest in the application of AI in recent years provokes reflection on the issue of students’ trust in this technology. In 2023, 74.0% of British young people aged 16 to 24 years used AI tools. The percentage of British teenagers aged 13–17 using AI was even higher (79.0%)^15^. In Poland, in 2023, more than two-thirds of students (68.0%) reported that they used or would use AI-based tools. What is more, nearly two in three students (60.0%) positively perceived the use of AI in education, which suggests growing trust in this technology in the near future^16^. In the global scale, the proportion of students consciously using AI tools in 2023 ranged from 43.0% ^17^ to 84.9% ^18^, and according to predictions the application of AI in the higher education sector will be increasing^19,20^.

There already is a considerable number of studies into the use of AI in the English-language context, but few of them focus on how AI users’ cultural background (including nationality, educational setting, and native language) may influence their interactions with this technology. This article attempts mainly to fill this knowledge gap.

The aim of the study was to identify the predictors of trust in AI among students in Poland and the UK. The study sought to determine the relationships between social and demographic factors and trust in modern technologies and to ascertain the differences in this respect between students in Poland and those in the UK. The choice of these countries was dictated not only by the fact that AI technologies analyze and generate text primarily based on the structure of the English language^21^ but also by differences in the level of technological transformation readiness. In Poland, the Information and Communication Technology (ICT) Development Index is 77.8, while in the United Kingdom, it is 80.9 ^22^. The higher score of the United Kingdom compared to Poland suggests that the UK has a more developed infrastructure, greater skills in new technologies, and a more innovation-friendly environment, which may also influence the differences in the level of trust in AI in these countries.

### Sociodemographic determinants of trust in AI: state of research

The literature indicates that trust in AI among students can be influenced by different sociodemographic variables, such as social background^23^, gender, age, and education level^24,25^. What also plays an important role is individual competencies, such as skills in using and understanding AI^25,26^, anthropomorphism, the attribution of human characteristics to AI^27,28^, students’ striving for independence in the process of education^29^, belief in the technical reliability of AI^27^, and appreciating the functionality, usefulness^30^, and innovativeness of AI^23^. Research conducted in various countries and educational settings also show that the way people perceive and trust AI can differ considerably. These differences are often linked with nationality^31^, the specific cultural and educational characteristics of a given setting^32–34^, the religion professed^35^, or the reported level of religiosity^36^. Table 1 presents a list of factors that influence students’ trust in AI tools based on a review of the literature.

**Table 1:** Factors influencing students’ trust in AI: a literature review.

Characteristic	Description	Authors
AI literacy	Students confident about their skills in using and understanding AI have a more positive attitude towards it, which contributes to their trust.	^26^
AI anthropomorphism	Attributing human characteristics to AI positively influences trust in these technologies.	^27^ ^28^
Individual predispositions	Students’ willingness to interact with AI, their perception of AI, and their need for autonomy in the process of education strengthen trust in AI.	^29^
Gender	Men trust AI more often	^25^ ^24^
Age	Trust generally increases with age.	^25^ ^24^
Education level	Greater trust in AI among individuals with higher education	^25^
Belief in the technical reliability of AI	Trust in the functions performed by AI increases with the belief in its reliability.	^27^
AI functionality and usefulness	Trust is related to the capability of AI to effectively perform the tasks entrusted to it, better interpretation of solutions, the reliability of AI operation, and the user-friendliness of its interface.	^29^
AI innovativeness	The perception of AI as a technology offering unique possibilities strengthens trust.	^23^
Expectations and experience	Differences between the expected evaluation and the evaluation received from an AI system influence students’ trust (receiving an evaluation better than expected from AI increases students’ trust in AI).	^30^
Cultural and educational context	Attitudes towards AI, including trust, may vary depending on the country and the educational context.	^32^ ^31,34^ ^33^
Religiosity level	Religious individuals more often showed intense emotions such as anger or fear towards AI.	^36^
Religions	Higher religiosity in the followers of historically dominant Christian religions was associated with lower AI anxiety.	^35^

### Framework

As the theoretical framework, we adopted the Unified Theory of Acceptance and Use of Technology (UTAUT 2 and meta-UTAUT), according to which sociodemographic factors play an important role as predictors of attitudes, including trust in AI^28^. According to the UTAUT, variables such as users’ gender, age, experience, and education level can significantly differentiate the way in which a technology is adopted and used^35^. Moreover, the results of previous studies indicate associations of religiosity level^37^ 36 and cultural determinants with the perception of AI^38,39^. This induced us to extend the UTAUT model to include factors such as nationality, educational setting, and religiosity. These seem to be relevant, particularly in the context of culturally diverse communities^40^. Nationality may therefore influence Performance Expectancy, as cultural differences can shape expectations regarding the effectiveness of technology and its ethical perception, which in turn relates trust in AI. Education may influence both Effort Expectancy and Performance Expectancy, as individuals with higher education are more likely to perceive AI as easier to use and more useful, which increases their trust. Religiosity may relateFacilitating Conditions, as religious individuals may have greater ethical concerns regarding AI, which could reduce their trust in this technology.

## Methods

### Research design

To collect data in Poland, we performed a cross-sectional study using respondent-driven sampling (RDS)^36,41,42^, which is one of the methods “designed to generate estimates that are representative of the wider population of interest, despite the biased sampling”^43^. Although RDS was originally designed for studying hard-to-reach populations, it now extends beyond its initial applications^44^. This method utilizes sample weighting based on participants’ network size, allowing for the correction of potential biases resulting from non-representative recruitment and achieving reliable population estimates^45^. For respondents from Poland, to ensure a representative sample, we applied limits based on the classification of fields of education and training according to ISCED-F 2013, published by the Central Statistical Office (GUS) in June 2023 ^46^. RDS, while not strictly a probabilistic method, by applying appropriate assumptions and techniques (such as sample weighting), successfully approximates results obtained through classical probabilistic methods^47^.

We also used the Savanta research panel with more than 150,000 students from the UK^48^. Panel respondents in the UK were recruited using the Universities and Colleges Admissions Service (USAC) database^49^. Representativeness was ensured by establishing representative quotas based on HESA population data (HESA, 2023), which included gender, year of study, and university group (including the Russell Group, pre-1992 universities, post-1992 universities, and others)^50^. This sampling technique provided a stable foundation for each demographic group, which is crucial for the reliability of the results.

### Procedure

The study was conducted in accordance with the requirements of the Declaration of Helsinki. Respondents were informed about the aim of the research and provided written informed consent at the beginning of the electronic survey. Participation in the study was anonymous and voluntary. The study was conducted using computer-assisted web interviewing (CAWI), a technique that makes it possible to reach a large group of respondents more easily and flexibly and increases anonymity and the sense of privacy, which—as research shows−leads to more honest and accurate answers, especially in the case of questions concerning sensitive topics^51^. Before the study began, respondents were informed that no personal data that could compromise their privacy would be collected, and only aggregate data would be used in further analysis. Respondents were also informed that data sharing would be possible only upon explicit request. The data are stored in a repository in the form of a statistical file, while the key code necessary to interpret respondents’ answers is deposited in a separate repository, providing an additional layer of security.

### Data collection

In the study conducted in late 2023 and early 2024, we used our own measure—a questionnaire of students’ attitudes towards AI. The approach to attitudes draws on Rosenberg and Hovland’s theory of attitudes^52^. One of the investigated dimensions of attitudes is trust, measured using a specific subset of items rated on a 5-point scale, where 1 means that the respondent strongly disagrees with a given statement and 5 indicates full agreement with the content of a particular item. The content of the items concerning trust in AI was designed based on the existing literature, which distinguishes three dimensions of trust in this technology (reliability, usefulness, and functionality)^27^ or discusses this issue more specifically, distinguishing categories of trust in new technologies^12^. The content of the items was related both to these dimensions and to the categories of trust in modern technologies and was intended to meet the measurement validity principle^53^ (Table 2).

**Table 2:** Operationalization of trust in AI through AI technology application scenarios.

Dimension	Lockey’s category	Item content
Reliability	Accuracy and reliability	Using medical services in which physicians are replaced by AI
	Automation	Changing to an automatic car
Functionality	Mass data extraction	Entrusting AI with performing financial transactions
	Experience and familiarity with the system	Entrusting AI with performing one’s professional duties
Usefulness	Anthropomorphism	Entrusting AI with childcare
	Transparency and explainability	Entrusting AI with activities in the domain of legal services

The overall trust in AI score, which is the sum of item ratings, ranges from 6 to 30. The higher the score, the more positive the attitude towards AI. The analysis of the measure yielded an acceptable value of Cronbach’s α for the subset of items (0.906). Additionally, an exploratory factor analysis was conducted using the Principal Components Analysis method and Varimax orthogonal rotation with Kaiser normalization. The KMO measure of sampling adequacy was 0.917, and Bartlett’s test of sphericity was significant (*p* < 0.001), confirming the suitability of factor analysis. High factor loadings (ranging from 0.795 to 0.852) indicate that all variables strongly correlate with a single common construct, further supporting the validity of the instrument.

### Statistical analysis

To identify the sociodemographic factors that would predict the results, we used multiple linear regression analysis (a stepwise method). The assumption regarding collinearity was tested through an analysis of the variance inflation factor (VIF). VIF values between 1 and 5 indicate that independent variables do not show strong collinearity and that they are independent of each other. This means they can be retained in the model with no fear of excessive intercorrelation relating the estimation of regression coefficients.

**Fig. 1:**
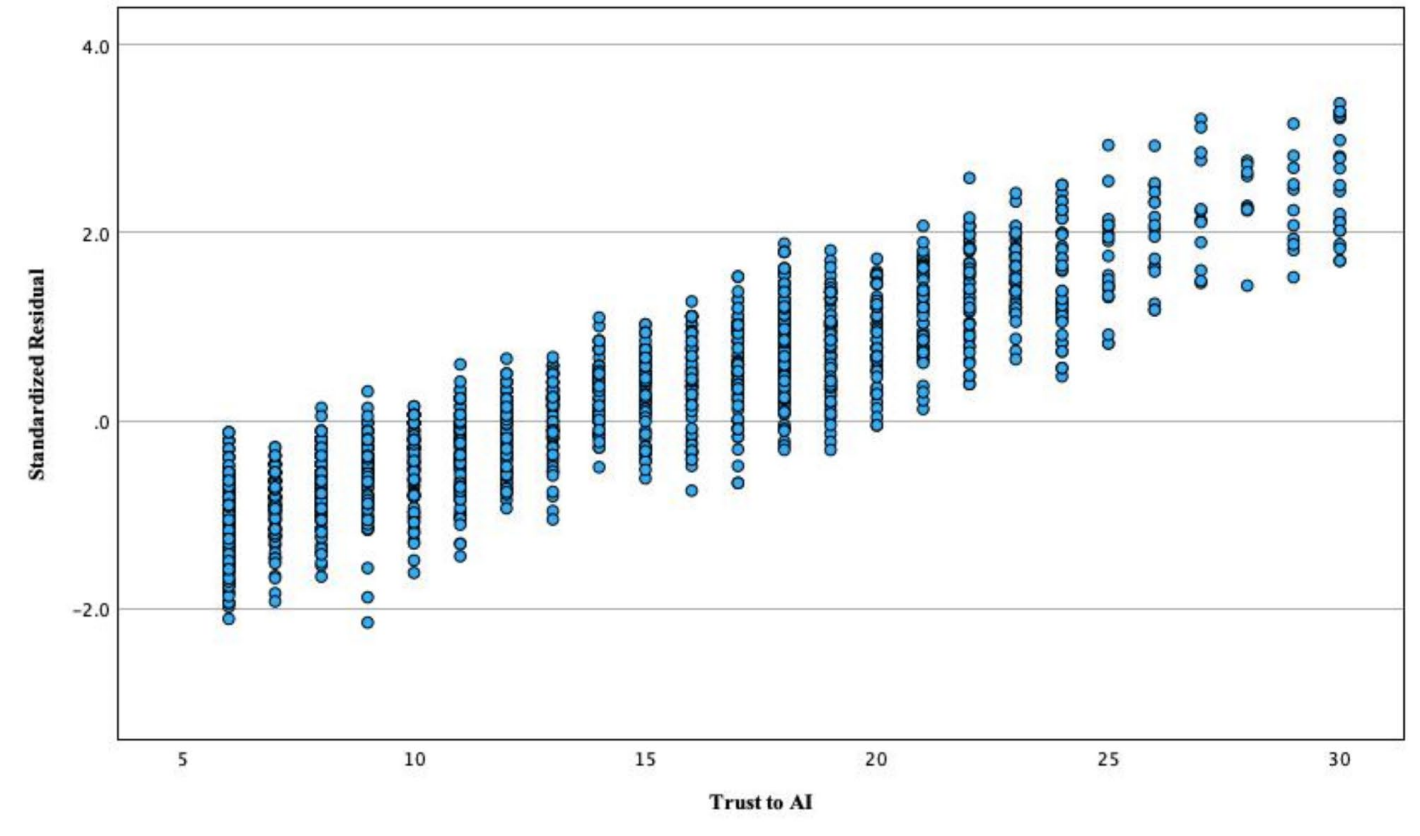
Homoscedasticity analysis based on standardized residuals and predicted values of the dependent variable.

To assess homoscedasticity, we used the analysis of standardized residuals in relation to the predicted values of the dependent variable (Fig. 1).

It was impossible to unambiguously determine if the homoscedasticity assumption was violated. Therefore, in further analysis we decided to use regression analysis with bootstrapping, which provides a more stable assessment of standard errors, resistant to some violations of the regression model, including heteroscedasticity. This makes it possible to generate the distribution of model estimators and, subsequently, to determine more reliable confidence intervals and assess the stability of model estimates^54^.

The two-way ANOVA was used to assess the level of trust in AI with the simultaneous impact of two classificatory factors. In our article, analysis is focused on interaction between gender and place of study.

### Institutional review board statement

The study was conducted in accordance with the Declaration of Helsinki and approved by the Humanities & Social Sciences Research Ethics Committee (HSSREC)—protocol code 12/2023; date of approval: 02/11/2023.

### Informed consent statement

Informed consent was obtained from all subjects involved in the study.

## Results

The mean score on trust in AI was 12.7 (SD = 6.3), which suggests a certain degree of diversity regarding the level of trust in AI. The mean level of trust in AI among Polish students was 11.6 (SD = 5.9), while British students’ mean trust was slightly higher (M = 14.0, SD = 6.4). The statistically significant difference between these two groups (t = − 8.930, *p* < 0.01) suggests that place of study had an effect on the level of trust in AI: those who studied in the UK trusted AI more strongly and would be more willing to entrust this technology with some functions or roles. Table 3 presents detailed data concerning the level of trust in AI.

**Table 3:** Analysis of trust in AI according to sociodemographic characteristics.

Variables		*N*	M	SD	Test score	*p*	Effect size
Students from…	PL	1088	11.6	5.9	− 8.930	*p* < 0.01	0.037
	UK	1010	14.0	6.4			
Gender	Male	666	14.8	6.5	57.099	*p* < 0.01	0.052
	Female	1350	11.7	5.8			
	Prefer not to say	82	12.4	6.4			
Religion	None	640	12.4	6.0	7.276	*p* < 0.01	0.007
	Christian	1081	12.6	6.3			
	Non-Christian	277	14.1	6.3			
Command of English	Poor	201	13.2	6.4	7.382	*p* < 0.01	0.007
	Average	446	11.7	5.7			
	Good	1451	13.0	6.3			
Religious practices	Non-practising	1157	11.7	5.6	36.528	*p* < 0.01	0.034
	Irregularly practising	424	13.8	6.4			
	Regularly practising	517	14.2	7.0			
Religious development	Upward trend	367	14.4	7.1	16.442	*p* < 0.01	0.015
	Downward trend	371	12.5	6.0			
	Stable level	1360	12.4	6.0			
Year of study	1–3 (younger students)	1718	12.3	6.0	− 6.239	*p* < 0.01	0.023
	4–5/6 (older students)	380	14.8	6.3			
Nationality	PL	927	11.0	5.6	109.663	*p* < 0.01	0.095
	UK	662	12.8	6.2			
	Other	509	15.9	6.3			

In the two-factor model, we analysed the effects of place of study and gender on trust in AI. The main effect of the analysed place of study on trust in AI turned out to be statistically significant (F = 4.620, *p* = 0.032) but low (η^2^ = 0.002). The main effect of gender on trust in AI showed statistical significance (F = 61.452, *p* < 0.001), and its strength was moderate (η^2^ = 0.055).

Moreover, the analysis revealed the statistical significance of interaction between place of study and gender (F = 7.214, *p* < 0.001), with an additional, relatively small, effect on trust in AI (η^2^ = 0.007).

The constant of the model, reflecting the baseline level of trust independent of the effect of other variables, showed high statistical significance (F = 1467.314, *p* < 0.001), with a partial η^2^ = 0.412. The value of R2 for the model was 0.096 (adjusted R2 = 0.094), which indicates that the model explained approximately 9.4% of the variance in the dependent variable.

**Fig. 2:**
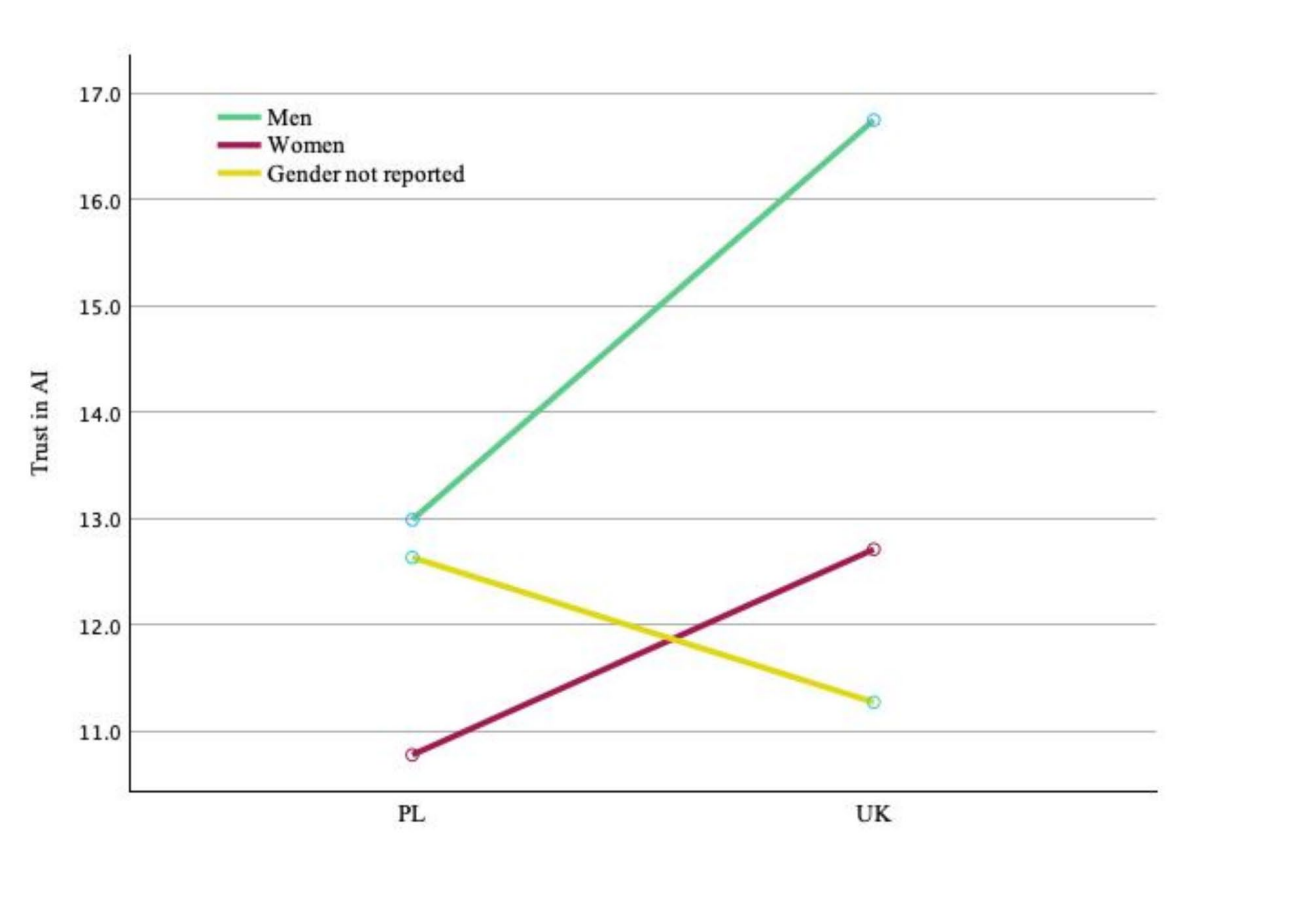
Effects of gender and place of study on trust in AI.

Male students, regardless of place of study, showed a higher level of trust in AI than female students or individuals who did not report their gender. Compared to those from Poland, both male and female students from the UK trusted AI more. In the case of individuals who preferred not to report their gender, there was a visible trend contrary to the one in the remaining respondents, with differences in trust in AI higher in Poland than in the UK (Fig. 2).

To identify the potential predictors of trust in AI, we performed a stepwise multiple linear regression. The final model, including six predictors, turned out to be statistically significant (F[6, 2091] = 68.795, *p* < 0.001), explaining 16.5% of the total variance in trust. The stepwise model revealed that the significant predictors were nationality, gender, year of study, place of study, frequency of religious practices, and religious development.

A more positive attitude of trust in AI was found in male students from the UK, those who had studied longer, practising religious believers, and individuals who experienced changes in their religious development.

The significant positive effect of nationality on trust in AI suggests that British people have higher trust in AI than Polish people and other nationalities. Beta coefficient indicates a moderate strength of this relationship.

Gender had a significant negative effect on trust in AI. Women and individuals who preferred not to reveal their gender had lower trust in AI than men. The value of Beta indicates a significant but non-dominant effect of gender on trust in AI.

Students in higher years showed higher trust in AI than those at the beginning of study. This suggests that length of study may influence the perception of and trust in AI.

Students studying in the UK had higher trust in AI than those studying in Poland. Cultural factors can have an effect on trust in new technologies.

Higher frequency of religious practices positively correlates with higher trust in AI, which may suggest that more religiously committed individuals may be more open or have a more positive attitude to technological innovations.

Individuals with a stable level of religious development had lower trust in AI than those whose religiosity showed an upward or downward trend, which is indicated by the negative Beta coefficient. This may indicate complex relationships between the dynamics of religious life and trust in AI. Detailed results of the analyses are presented in Table 4.

**Table 4:** Results of multiple regression analysis for the “trust in AI” outcome variable in the final model.

Model		Unstandardized coefficients		Beta	t	*p*	Tolerance	VIF
		B	S.E.					
Nationality	0 = PL; 1 = UK	0.355	0.037	0.210	9.672	*p* < 0.001	0.849	1.18
Gender	0 = Male; 1 = Female	− 2.08	0.238	− 0.175	− 8.738	*p* < 0.001	0.991	1.01
Year of study		1.789	0.33	0.111	5.450	*p* < 0.001	0.970	1.03
Students from…	0 = PL; 1 = UK	1.641	0.268	0.131	6.114	*p* < 0.001	0.871	1.15
Religious practices	1 = low; 5 = high	0.497	0.100	0.131	6.114	*p* < 0.001	0.858	1.17
Religious development	1 = low; 3 = high	− 0.497	0.167	− 0.06	− 2.973	*p* < 0.001	0.935	1.07

To sum up, trust in AI increased with the length of study and the frequency of religious practices; it was higher in men than in women and in British respondents than in Polish ones. Individuals with an increasing or decreasing level of religiosity showed higher trust in AI than those with a stable religiosity level, which suggests that the dynamism of changes in religiosity level may favour openness to new technologies.

## Discussion

The aim of our study was to determine the relationship between sociodemographic factors and the level of trust in AI among students in Poland and the UK and to identify the differences between these groups (trust was defined as having three dimensions (reliability, functionality, and usefulness) and was operationalized as a single construct—a scale). In investigating this issue, we applied the UTAUT model; the results of our study supported its assumptions. This corresponds with previous findings reported by numerous authors, concerning the associations of factors such as gender^55^, age^21^, and experience^56^ with attitudes towards AI.

In our study, we found a significant relationship between age and trust in AI. Men showed higher trust than women, which supports earlier reports present in the literature about gender differences in the perception of technology. This finding is reflected in publications by many authors who investigated attitudes in groups such as medicine students to AI^57^. There are suggestions in the literature that women, usually more cautious and inclined to avoid risk, express greater security and privacy concerns associated with the acceptance of new technologies^58^. Additionally, women may be more sensitive to potential health risks associated with new technologies^59^.

In our study, we found that students in higher years of study trusted AI more than less advanced students did. The literature provides support for this finding. An explanation of this fact is sought in the approach to the new reality becoming increasingly open with the knowledge gained, which allows individuals to better understand the value and potential of AI^60^. Their longer experience as students may translate into a more highly developed ability to see the potential benefits of innovation in the process of education. In addition, students in the upper years may be more likely to adapt new technologies as they have had fewer negative experiences with online education during the Covid-19 pandemic^61^. As a result, they become more open to change based on previous positive experiences with modern technology, which in turn translate into a more balanced approach to the evaluation of new technological tools, such as AI^62^.

We assumed that the basic set of variables in the UTAUT required complementing and added further variables: nationality, place of study, religious practices, and religious development. We found that they were significantly related to the level of trust in AI. This practice of expanding the UTAUT model by incorporating new variables has been acknowledged in the literature, indicating its adoption by other researchers and potential to produce valuable insights^32^. As shown in the literature, incorporating additional variables, such as environmental and psychological factors, significantly enhances the predictive power of models regarding user behaviour towards new technologies^63^. The results of our research support the thesis that, in the student environment, there are other social and demographic factors, unexplored by researchers so far, that influence trust in new technologies. Our finding indicate the need for identifying other factors that may play a role in the development of trust in AI.

In our study, we found that British students showed higher trust in AI than Polish ones. Differences in trust in AI between students of different nationalities have been confirmed in the existing studies. The analysis performed by Strzelecki and ElArabawy revealed, based on the use of ChatGPT in Egypt and Poland as an example, that acceptance of AI technologies depended not only on access to new solutions and on their usefulness but also on the cultural, social, or educational. The authors point out that technological infrastructure and the approach to education in the use of new technologies can significantly differentiate the level of trust in AI. This corresponds with our findings, where British students demonstrated higher levels of trust^34^. Greater trust in AI in the UK compared to Poland may stem from differences in technological infrastructure, access to modern tools, or the emphasis on technological education within the higher education system. In 2023, the UK led in the availability of AI-related degree programs, offering more courses in this emerging technology than the USA, despite having fewer universities. The UK offered 744 AI programs in English, while the United States had 667 ^64^.

The analysis of results concerning place of study in the context of trust in AI also yielded interesting findings. Scores on trust in AI were higher among respondents studying in the UK. It can be supposed that this pattern may partly stem from language differences, particularly in the context of proficiency in English^65^. Students who use this language freely may have better access to the latest AI solutions, which in turn may contribute to building greater trust in these technologies. Additionally, the fact that many AI systems, including popular linguistic models such as ChatGPT, are developed mainly based on the English language may also influence the perception of the usefulness and reliability of AI, which may differentiate trust in new technologies^66^.

Research results also reveal the role of religiosity in developing trust in AI, showing that individuals with higher levels of religiosity, as indicated by factors such as frequency of religious practices and self-reported religiosity, more willingly express trust in these technologies^36^. On the other hand, individuals maintaining a stable level of religious development exhibit lower trust in AI compared to those whose religiosity does not change. It can therefore be said that the phenomenon of religiosity is a significant factor in the shaping of reality, including the developing AI technology. This points to the need to include religious aspects in the theoretical framework of the UTAUT, which will contribute to a better understanding of how religious beliefs and the related interiorized values shape attitudes towards AI. In other words, apart from the classic predictors of trust in AI, religious and cultural aspects also play a key role in the acceptance of new technologies, which opens new research and theoretical perspectives. The need to expand the UTAUT framework to include elements such as religiosity in the context of trust in technological innovations is voiced as a recommendation, for example, among researchers in the field of economics ^67,68^.

To sum up, the results of our research support and extend the findings reported in the literature concerning the factors significantly related to trust in AI, thus attesting to the complexity and multidimensionality of this issue.

## Conclusion

The presented research results concerning the sociodemographic determinants of trust in AI open prospects for further research in this dynamically developing area. They suggest the possibility of further expanding the UTAUT theory by the inclusion of new social and demographic factors, such as educational setting and religiosity, which may play a role in the development of trust in AI.

Moreover, research results indicate differences in the level of trust in AI between students from different countries, suggesting a link between the cultural and educational context and attitudes towards new technologies. This points to the need for broader comparative research into the effect of different cultural settings and educational systems on the perception and acceptance of AI.

In the context of globalization and the dominance of English in technology and science, there seems to be a need for further research into the role of language in the acceptance of AI. It can serve to determine the associations of the multilingualism and local adaptations of AI systems with the trust placed in them and with the acceptance they enjoy in various communities. The results of future studies that would include these aspects associated with the development and implementation of AI may contribute to societies taking a balanced approach to this technology: avoiding both “blind trust” and unfounded negative emotions towards it^69^.

Future studies in this field should seek a holistic approach, covering both technological and sociocultural aspects of acceptance and trust towards AI.

### Limitations

There are several limitations that should be taken into account when interpreting the results of our study. It is important to emphasize that the findings of this study pertain exclusively to students from Poland and the United Kingdom, limiting the ability to generalize the results to other age, professional, and cultural groups. Students, as a specific social group, may perceive artificial intelligence differently compared to individuals from other professional backgrounds, such as corporate employees or older adults, whose daily experiences with technology may significantly vary. Therefore, further research involving more diverse samples is necessary to validate these conclusions in other social and cultural contexts. Additionally, as the study only covered European countries, future research could expand the sample to include students from regions such as the Middle East or Asia to gain a more comprehensive insight into the sociodemographic predictors of trust in AI. It is also worth considering broadening the comparative perspective, for example using Hofstede’s cultural dimensions, to better understand the importance of cultural factors, which could explain why UK students show higher levels of trust. Additionally, the presence of religiosity as a predictor of trust in AI suggests a specific influence of cultural and religious values on attitudes toward new technologies, particularly artificial intelligence. It is worth considering differentiating between regular and irregular religious practices to better understand how they relate to risk perception and ethical issues with trust in AI.

Lastly, the scale used to operationalize trust in artificial intelligence, although based on three dimensions (reliability, functionality, and usefulness), was treated as a unidimensional construct, which may relate the full capture of the complexity of participants’ attitudes. Future research should consider a more detailed approach to operationalizing this construct, which could provide even deeper insights. It should also be noted that the study design is based on correlational analysis, which means that it does not allow causal conclusions to be drawn.

## Data Availability

The raw data supporting the conclusions of this article will be made available by the authors on request. Please contact Jarosław Kozak at jaroslaw.kozak@kul.pl to request the data.
